# Face Recognition Characteristics in Patients with Age-Related Macular Degeneration Determined Using a Virtual Reality Headset with Eye Tracking

**DOI:** 10.3390/jcm13020636

**Published:** 2024-01-22

**Authors:** Nina Žugelj, Lara Peterlin, Urša Muznik, Pia Klobučar, Polona Jaki Mekjavić, Nataša Vidović Valentinčić, Ana Fakin

**Affiliations:** 1 Eye Hospital, University Medical Centre Ljubljana, 1000 Ljubljana, Slovenia; nina.zugelj84@gmail.com (N.Ž.); peterlinlara@gmail.com (L.P.); ursa.malesic@kclj.si (U.M.); pia.klobucar@kclj.si (P.K.); polona.jaki-mekjavic@mf.uni-lj.si (P.J.M.); natasa.vidovic@kclj.si (N.V.V.); 2 Faculty of Medicine, University of Ljubljana, 1000 Ljubljana, Slovenia

**Keywords:** virtual reality, AMD, face recognition, eye tracking, heatmap, fixation

## Abstract

Background and Objectives: Face recognition is one of the most serious disabilities of patients with age-related macular degeneration (AMD). Our purpose was to study face recognition using a novel method incorporating virtual reality (VR) and eye tracking. Materials and Methods: Eighteen patients with AMD (seven male; median age 83 years; 89% with bilateral advanced AMD) and nineteen healthy controls (five male; median age 68 years) underwent the face recognition test IC FACES (Synthesius, Ljubljna, Slovenia) on a VR headset with built-in eye tracking sensors. Analysis included recognition accuracy, recognition time and fixation patterns. Additionally, a screening test for dementia and imaging with fundus autofluorescence and optical coherence tomography was performed. Results: AMD patients had significantly lower face recognition accuracy (42% vs. 92%; *p* < 0.001) and longer recognition time (median 4.0 vs. 2.0 s; *p* < 0.001) in comparison to controls. Both parameters were significantly worse in patients with lower visual acuity. In both groups, eye-tracking data revealed the two classical characteristics of the face recognition process, i.e., fixations clustering mainly in the nose–eyes–mouth triangle and starting observation in the nasal area. Conclusions: The study demonstrates usability of a VR headset with eye tracking for studying visual perception in real-world situations which could be applicable in the design of clinical studies.

## 1. Introduction

Age-related macular degeneration (AMD) is an acquired disease of the macula characterized by progressive visual impairment because of late-onset neurodegeneration of the photoreceptor–retinal pigment epithelial complex. AMD is a major cause of central visual loss and affects 10% of people older than 65 years [1]. AMD may be divided into various stages [2]. Early and intermediate AMD are characterised by yellow/white deposits (drusen) beneath the retinal pigment epithelium and/or areas of focal hyperpigmentation or hypopigmentation. The advanced stage may take one of two forms: neovascular AMD, characterised by growth of new blood vessels beneath the retina with a tendency to leak, causing sudden vision loss, or geographic atrophy (GA), characterised by sharply demarcated areas of hypopigmentation caused by atrophy, causing more insidious vision loss. Neovascular AMD is often referred to as ‘wet’, whilst non-neovascular AMD (i.e., early and intermediate AMD and GA) may also be known as ‘dry’ AMD and constitutes about 90% of diagnosed cases of AMD [3]. Patients suffering from this process have an important reduction on their quality of life being handicapped to read, to write, to recognise faces of their friends, or even to watch the television [4]. Face recognition is one of the most important functions of our visual system. The ability to recognise familiar faces is important in many settings. A person’s identity, gender, age, and emotion can be recognised from even a brief glance at their face. This information is useful to navigate both social and professional settings [5]. Indeed, disorders of face processing are associated with social anxiety [6,7] and substantial difficulties in the work place [7]. Face recognition has been studied previously in healthy people and in association with various diseases. Studies have shown that most viewers preferentially fixate in the triangle of the eyes, nose, and mouth [8,9,10,11,12,13]. Hsiao et al. showed that optimal recognition performance is achieved with the first two fixations and that the first and most important fixation is usually in the nasal region [9]. It has been suggested that individuals with autism spectrum disorders (ASDs) experience difficulties in facial recognition as a consequence of different strategies in facial processing [14]. Face recognition is significantly affected in patients with frontotemporal dementia (FTD) [15]. Glaucomatous patients with more advanced visual loss performed worse in a test of face recognition than patients with milder defects and people with healthy vision of a similar age [16]. People with AMD have difficulty with different aspects of face recognition [3]. For example, in a survey of 30 people with bilateral AMD, all but one reported difficulty recognising familiar faces on the street; a third of these felt embarrassment as a result [17]. In the same study, over half of respondents reported missing things in conversation because of an inability to make out facial expressions. These patient-reported data are corroborated by performance-based research studies. For example, viewing distances required for recognising faces were found to be shorter on average for people with AMD than those without [18]. Moreover, the ability to determine whether or not a face is expressive has been reported to be closely related to near reading acuity [17]. Patients with AMD do not typically have problems recognising faces until the disease is in its later stages, whereas patients with bilateral advanced AMD are more likely to experience them [3]. Patients with AMD are also at risk of dementia or specifically Alzheimer’s disease, which calls for greater clinical awareness of the comorbidity of both conditions among elderly people [19], especially since patients with dementia have also been shown to have a facial memory deficit [20]. Although it is well documented that people with AMD have difficulty with face recognition, it is challenging to envisage how difficult the task truly is. Previous research focused mostly on recognition accuracy [3,17,21], while only a few studies looked at recognition time [8,21] and fixation patterns [8,22]. The purpose of this study was to determine the characteristics of face recognition in patients with AMD by using a novel system that combines virtual reality (VR) with eye tracking.

## 2. Materials and Methods

### 2.1. Study Subjects

The study included 18 patients with visual loss due to AMD (7 male, 11 female) and 19 control participants with no ocular disease (5 male, 14 female). Inclusion criterion for both groups was age >50 years. AMD patients were recruited from the cohort of AMD patients that were under the care of the National centre for comprehensive visual rehabilitation of blind and visually impaired at the Eye Hospital, University Medical Centre Ljubljana, Slovenia. The inclusion criteria were the diagnosis of AMD determined by a specialist of Ophthalmology with an exam that included imaging with optical coherence tomography (OCT), AMD being the main reason for the visual loss, and best corrected visual acuity (BCVA) of ≤0.7 Snellen decimal (0.15 logMAR) on the better eye. Furthermore, since the face recognition test is performed without refractive correction, the inclusion criteria included a refraction error of ≤3.00 spherical equivalent and the difference between uncorrected VA (VA) and BCVA of ≤2.00 Snellen decimal. Inclusion criterion for the controls was uncorrected visual acuity (VA) on the better eye of ≥0.6 (0.22 logMAR).

### 2.2. Face Recognition

The study subjects underwent face recognition testing using the IC FACES software v. 1.0 (Synthesius, Ljubljana, Slovenia). The test consisted of faces of 13 famous people (see Appendix A) that were presented sequentially on a VR screen (HP Reverb G2 Omnicept Edition, Hewlett Packard, Spring, TX, USA) at a virtual distance of 1 m. The faces were placed on a squared cropped background, extending 28° of the visual field, while the faces themselves extend in diameter approximately 20° of the visual field, corresponding to the macular area. The test was performed binocularly and without correction. Each face was shown until the participant verbally confirmed recognition or for a maximum of 10 s, and the researcher entered the result (Recognized or Not recognized) in the accompanying software application on the computer. To avoid delay, the verbal confirmation was accepted as soon it was clear that the person was recognized (e.g., “Oh, that famous skier!”), and the full name and surname could be delivered afterwards. The analysis included the number of recognized faces and time to recognition. The VR headset also collected eye-tracking data, which was gathered from 15 AMD patients and 16 controls and analysed using IC FUSION software v.1.2 (Synthesius, Ljubljana, Slovenia). Heatmaps of fixation points were used to qualitatively assess the distribution of fixations and to determine the existence of internal and external fixations within each face. A representative face was used to determine the sequence of the first five fixations.

### 2.3. Visual Acuity and Imaging

Visual acuity was determined using Snellen charts in decimal annotation and converted to logMAR. Each eye was tested separately in AMD patients with and without correction, while visual acuity in control group was measured binocularly without correction. Visual acuity of counting fingers at 1 m was converted to 0.015, counting fingers at 30 cm to 0.010 and hand motion to 0.005 Snellen decimal. Patients also underwent imaging with fundus autofluorescence (FAF, *n* = 14) and optical coherence tomography (OCT, N = 18) (Spectralis, Heidelberg, Germany). AMD stage was determined for each eye of AMD patients according to previously established criteria [2]. For eyes with geographic atrophy (GA), its horizontal diameter was measured on FAF image using Spectralis software v. 3.1 and converted to degrees using the conversion of 1 mm of retina to 3.5° of the visual field. Two representative patients underwent a fixation test on the better-seeing eye using the fundus-controlled microperimeter (MP-2, Nidek Technologies, Padua, Italy). Results of the fixation test were exported on colour fundus images and superimposed on the FAF images using Adobe Photoshop (Adobe Inc., San Jose, CA, USA).

### 2.4. Dementia Screening

Since dementia occurs more frequently in patients with AMD [19], and could affect face recognition [20], a subgroup of AMD patients additionally underwent a screening test for dementia to assess the scale of this effect (*n* = 12). The test used was MoCAa-Blind (Montreal Cognitive Assessment, available at mocacognition.com, accessed on 6 July 2022). A numerical score from 0 to 22 was determined for each patient, with a lower score indicating a higher risk for dementia.

### 2.5. Statistical Analysis

Statistical analysis was performed using SPSS software version 27 (IBM SPSS Statistics; IBM Corporation, Chicago, IL, USA). Distribution of continuous variables between the two groups was tested using Mann–Whitney U test. Correlations between face recognition accuracy/recognition time and various parameters was assessed using Pearson correlation and multiple linear regression. Correlation between the use of fixations on external facial features and face recognition was assessed using Fisher’s Exact Test and multiple logistic regression. The less affected eye was determined based on visual acuity for each patient as it was thought that this eye was most responsible for face recognition at binocular viewing. In case of symmetrical visual loss, the eye with the smaller diameter of the atrophic lesion was selected.

## 3. Results

The demographic characteristics, clinical data, and the results of face recognition testing of AMD patients are shown in Table 1. The median ages of the AMD and control group were 83 years (range, 68–91) and 68 years (range, 54–87), respectively. The difference in age was statistically significant (Mann–Whitney U test, *p* < 0.001).

### 3.1. Visual Acuity

In the AMD group, the median uncorrected VA on the less affected eye was 0.80 logMAR (range, 0.30–2.00), and the median BCVA was 0.70 logMAR (range, 0.15–2.00). The median average interocular uncorrected VA was 1.25 logMAR (range, 0.35–2.00), and the median interocular difference was 0.90 logMAR (range, 0.00–1.40). Uncorrected VA on the better-seeing eye was significantly correlated with age (Pearson correlation, R = 0,48, *p* < 0.05). The median uncorrected VA of the control group was 0.02 logMAR (range, 0.00–0.22).

### 3.2. AMD Staging

The majority of AMD patients (16/18, 89%) had bilateral advanced AMD. One patient had bilateral intermediate AMD, and one patient had a combination of advanced and early AMD (Table 1). Spared foveal photoreceptors on the better-seeing eye were observed in 33% (6/18) of cases, among whom three had dry and three had wet AMD. These patients had significantly better VA (median 0.35 vs. 1.00 logMAR, Mann–Whitney U test, *p* < 0.01).

### 3.3. Face Recognition Accuracy and Time

**Figure 1:**
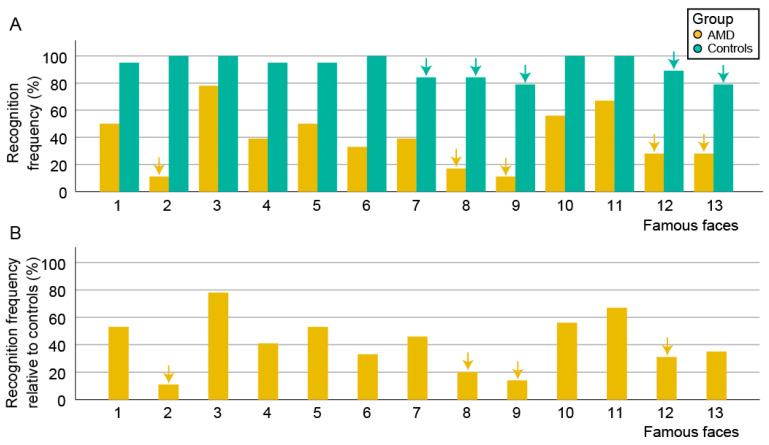
(**A**) Bar chart showing the percentage of participants from both groups that recognized each famous face. The five least often recognized faces within each group are marked with arrows. The four faces that were the least recognizable for both groups (8, 9, 12, and 13) are outlined with a red border. (**B**) Bar chart showing the frequency of face recognition in AMD group normalized to the recognition frequency of the control group. The four least recognizable faces (2, 8, 9, and 12) are marked with arrows and outlined with a red border. Note that faces 2 and 12 belong to people who became famous relatively recently, potentially after visual loss in some AMD patients.

**Figure 2:**
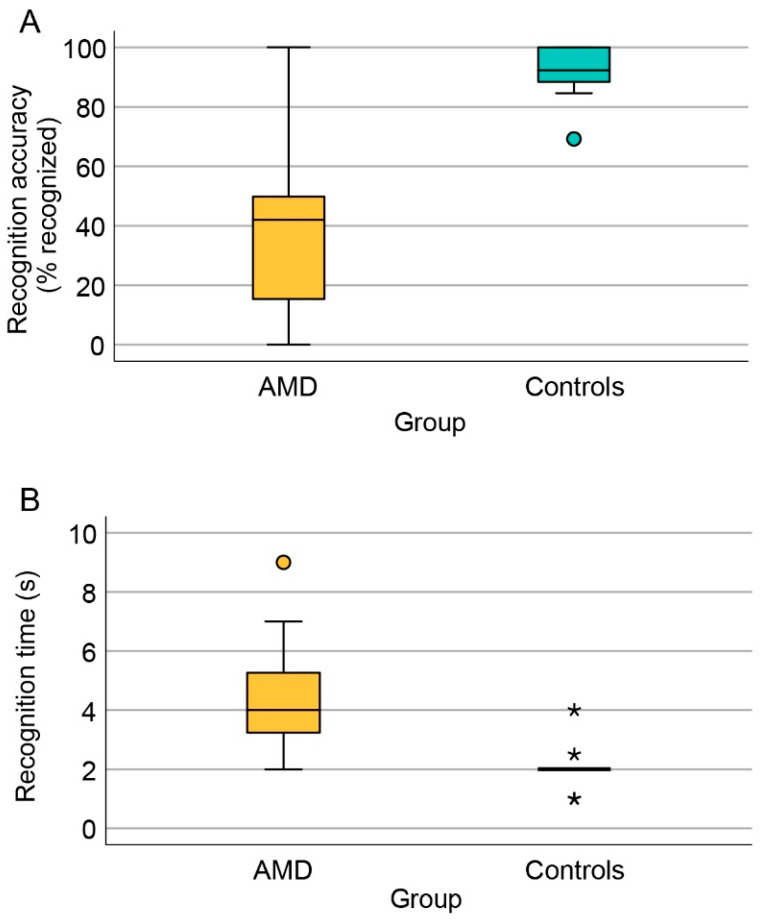
Recognition accuracy and time. (**A**) Boxplot chart showing the percentage recognized out of 13 famous faces. (**B**) Boxplot chart showing the distribution of median recognition times from each patient. If 10 s elapsed before recognition, the face was marked as “Not recognized”, and recognition time was not recorded. Outliers are marked with o and *, respectively.

The proportion of AMD patients and controls who recognized each famous face is shown in Figure 1. Control subjects were able to recognize a median of 92% of faces (69–100%) with 95% of them recognizing ≥11/13 (85%) faces, confirming the recognizability of the selected famous faces. Their median recognition time was 2.0 s (range, 1.0–4.0 s). AMD patients were able to recognize a median of 42% of faces (range, 0–100%), with 95% of them recognizing ≤ 11/13 (85%) faces. Their median recognition time of 4.0 s (range, 2.0–9.0 s. Both parameters were significantly worse in comparison to the control group (*p* < 0.001 for both) (Figure 2). Face recognition accuracy and time were not correlated with age or sex.

While age did not appear to be a factor significantly affecting recognition in the selected cohorts, we performed an additional analysis on subjects in subgroups with the same age range of 68–87 years to further inspect this potential confounding factor. After the exclusion of outliers, 14 AMD patients and 11 controls with median ages of 79 and 75 years remained (*p* = 0.13). Their median recognition accuracies were 46% and 92%, respectively, and the median recognition times were 4.0 and 2.0 s, respectively. Both parameters remained significantly different between the two groups (*p* < 0.01 for both).

There was some variation in the recognizability of individual famous faces between AMD patients and controls (Figure 1). The face with the largest discrepancy (Face 2), recognized by all controls but only two AMD patients, belonged to a person that has become famous relatively recently, potentially after the visual loss in some AMD patients, which could pose a problem in the methodology. A comparison of the recognition accuracy between the groups was therefore repeated after excluding this face; however, it did not show any difference (median 42% and 92%, respectively; *p* < 0.001). To further explore the extent of this potential bias, we excluded the other famous person that has also become famous relatively recently (Face 12). After this, the median recognition accuracies were 45% and 91%, respectively, but remained significantly different (*p* < 0.001). All other famous people had been famous for >10 years.

### 3.4. Correlation between Face Recognition and Visual Acuity

**Figure 3:**
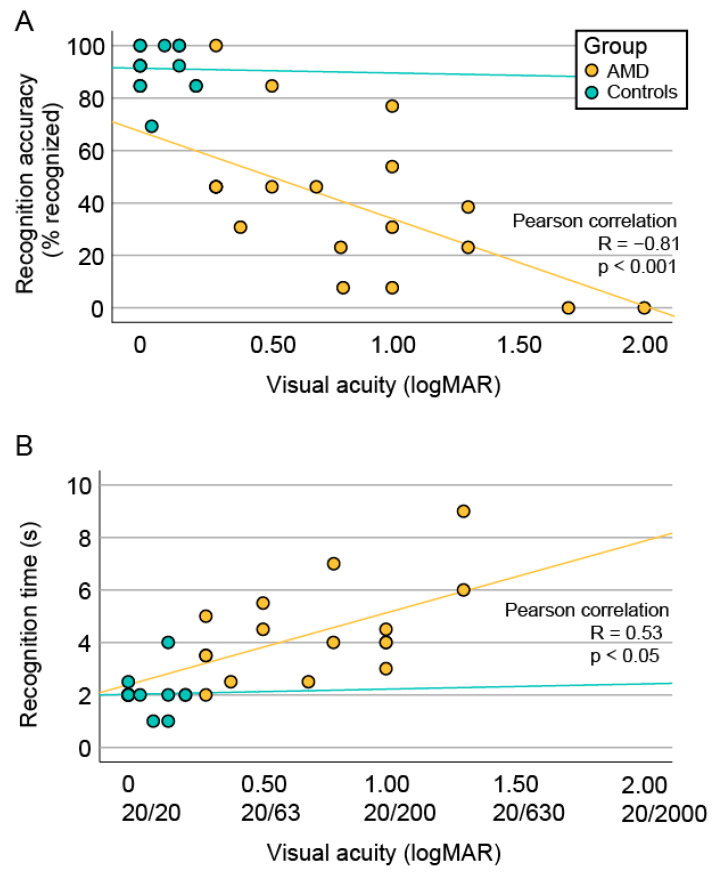
Correlation between face recognition and visual acuity. (**A**) Scatter plot showing the percentage of recognized faces in correlation with uncorrected visual acuity of the better-seeing eye. (**B**) Scatter plot showing the distribution of median recognition times from each patient in correlation with uncorrected visual acuity of the better-seeing eye. Pearson’s correlation coefficient is shown for AMD patients in whom the correlation was significant. Metric annotation of visual acuity is written below the logMAR values. Note that the patients who did not recognize any faces had no recorded recognition time.

In the AMD group, there was a significant correlation between visual acuity on the better-seeing eye and both recognition accuracy (R = −0.81, *p* < 0.001) and recognition time (R = 0.53, *p* < 0.05) (Figure 3).

### 3.5. Correlation between Face Recognition and Structural Biomarkers

The two patients with early or intermediate AMD on at least one eye had notably better recognition accuracy than 16 patients with bilateral advanced AMD (median 73% vs. 35%, respectively); however, the difference was not significant. Significantly higher face recognition accuracy was observed in 6/18 patients with preserved foveal photoreceptors (46% vs. 27%, *p* < 0.05). In those with GA, there was a trend of higher recognition accuracy in association with a smaller GA diameter on the better-seeing eye; however, the association was not significant (Pearson correlation R = −0.45, *p* = 0.20).

### 3.6. Eye Tracking Data

#### 3.6.1. Fixation Heatmaps

**Figure 4:**
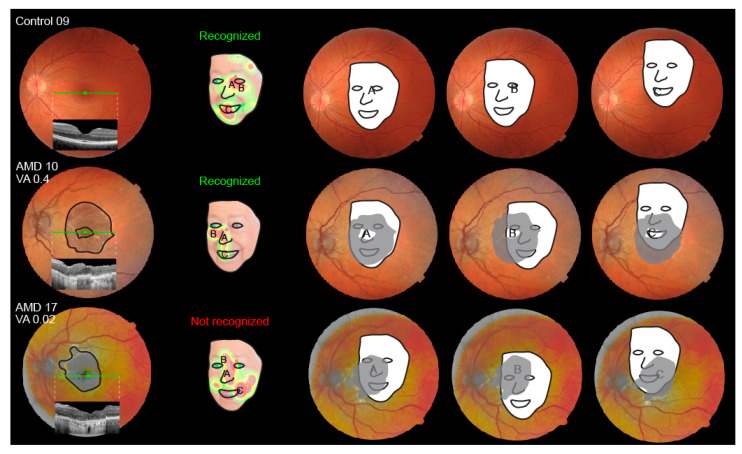
Schematic representation of the projection of a testing face on the retina for a control participant (top row) and two AMD patients (bottom two rows). Fundus image and OCT through the macula are shown in the first column. Fixation heatmaps are shown in the second column. The projection of the face on the retina is shown in the last three columns: three fixation areas, marked with letters (A–C) on the heatmaps. Note better visual acuity and spared foveal photoreceptors in patient AMD 10 (middle row), who was able to recognize the presented face. The photo of the famous patient was intentionally modified (cut out and reduced in contrast) to preserve anonymity for this figure.

A schematic representation of the approximate position of a testing face shown on the retina is shown in Figure 4. Qualitatively, the fixations of AMD patients were similar to those of the controls, located mainly in the triangle of the nose, eyes, and mouth. Besides that, there was a notable variability in individual heatmaps in both groups.

#### 3.6.2. Initial Five-Fixation Sequence

**Figure 5:**
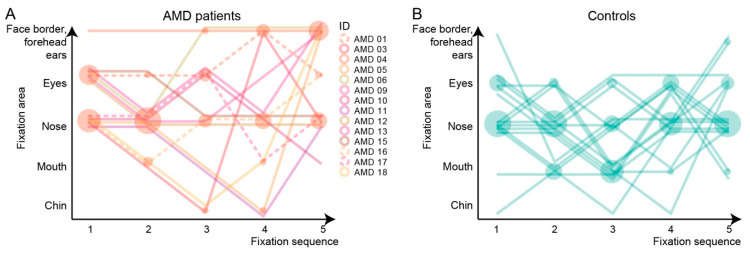
The sequence of the initial five fixations on a representative face (Face 3). (**A**) Fixations of AMD patients. (**B**) Fixations of control subjects. Fixations of each participant are connected with a line, which is dashed if the face was not recognized. The number of participants that fixated on each area is represented by the relative size of the filled circle at each position. Note that in both groups, participants most often fixated on the nose, which was also the most frequent starting point. Besides that, AMD patients relatively more frequently fixated on the external features (face border, forehead, ears, and chin) than controls.

The initial five-fixation sequences were determined by following the fixation traces using the IC FUSION module on a representative face (Face 3). The sequences are displayed graphically in Figure 5 and the proportions of participants who fixated on specific facial areas are listed in Table 2. In both groups, the nasal area was the most prevalent starting fixation, noted in 50% and 50% of AMD and controls, respectively, and at least once within the first two fixations in 79% and 75% cases, respectively (*p* > 0.05). Among the specified facial areas, there was a significant difference in the fixation frequency in the area that included the face border, forehead, and ears; noted in 57% of the AMD group and in 13% of the control group (Mann–Whitney U test, *p* < 0.05).

#### 3.6.3. Fixations on the Internal vs. External Facial Features

**Figure 6:**
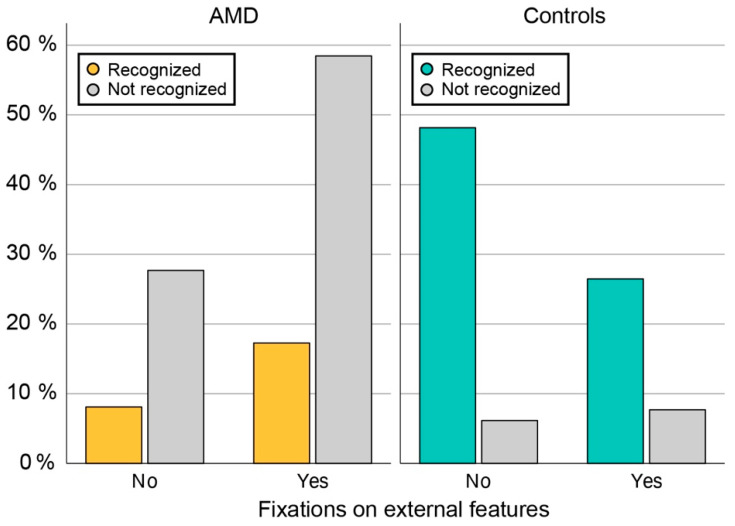
The proportion of recognized faces in subgroups of participants utilizing or not utilizing external fixations. AMD patients more often fixated on the external facial features than controls; however, this was not associated with increased face recognition accuracy.

The areas of interest were grouped into internal features (eyes, nose, mouth) and external features (face border, forehead, ears, and chin), to examine whether there was a significant difference in fixations on these regions between the two groups. Qualitative analysis of all heatmaps was performed to identify the cases where fixation on external features was utilized. External fixations were utilized significantly more frequently by AMD patients (on a median of 77% of faces; range, 15–100%) in comparison to controls (median 38%; range, 0–85%; Mann–Whitney U test, *p* < 0.01). We aimed to determine if there was a correlation between the presence of external fixations and recognition accuracy in the AMD group. The proportions of recognized faces in subgroups utilizing or not utilizing external fixations are shown in Figure 6. There was no difference in face recognition accuracy of AMD patients who had or had not utilized external fixations (Fisher’s Exact Test, *p* = 0.55) (Figure 6). Multiple logistic regression was performed to include the variable of visual acuity and showed a significant association between recognition accuracy and visual acuity (Exp(B) = 0.1, *p* < 0.001) but no association with external fixations (*p* = 0.86).

### 3.7. Shift of the Preferential Retinal Locus

**Figure 7:**
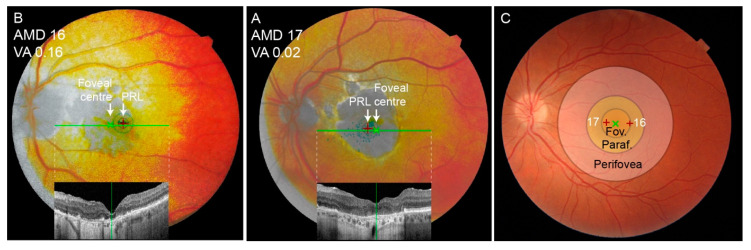
(**A**,**B**) Location of the PRL of the better-seeing eye of two AMD patients. Green crosses mark the PRL location and red crosses mark the foveal centre, identified with the help of OCT. Blue dots mark the fixation loci. ID and Snellen decimal visual acuity of each patient is noted in the top left corner. (**C**) The location of the fovea, parafovea, and perifovea marked with circles on a representative fundus image from the Eye Hospital Ljubljana archive. The fovea and the PRL of both patients are marked with green and red crosses, respectively. Note that both patients fixated within the foveal area. PRL—preferred retinal locus; Fov.—fovea; Paraf.—parafovea.

Fixation test with a fundus-controlled microperimetry device was performed in two representative patients on their better-seeing eye to determine the shift of their preferential retinal locus. The fixation results superimposed on the FAF image are shown in Figure 7. Both patients exhibited a PRL shift to an area within the foveal region.

### 3.8. Dementia

**Figure 8:**
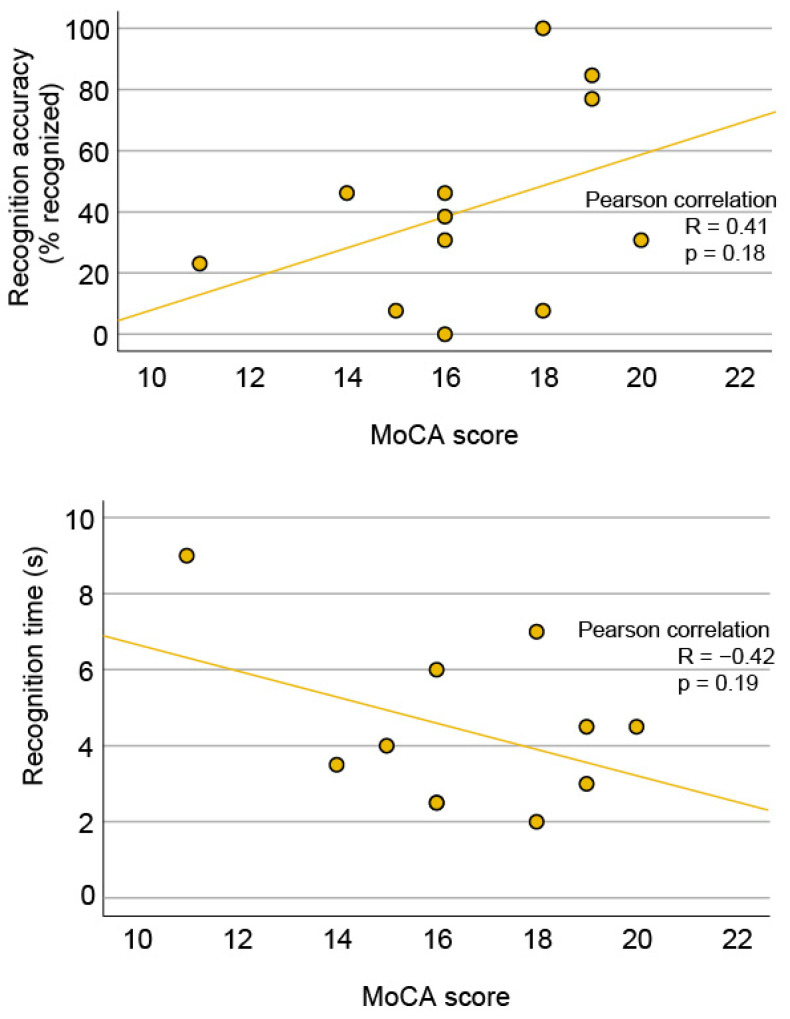
Correlation between face recognition and modified MoCA score in patients with AMD. Lower MoCA score indicates a higher risk for dementia. MoCA—Montreal Cognitive Assessment.

The median MoCA score of AMD patients was 16 points (range, 11–20). There was a trend of lower face recognition ability in patients with a lower score and thus a higher risk for dementia, for both, recognition accuracy (R = 0.41) and recognition time (R = −0.42); however, the correlations were not statistically significant (Figure 8). There was no correlation between the MoCA score and visual acuity, age, or lesion diameter.

## 4. Discussion

This study used a novel system combining VR with eye-tracking sensors to gather insights into face recognition ability of AMD patients.

### 4.1. Degree of Impaired Face Recognition in AMD Patients

The results confirmed that face recognition is a major issue for AMD patients, especially those with the advanced stage, who represented 89% of the study cohort. The median recognition accuracy of AMD patients was approximately twice as low in comparison to controls (42% vs. 92%, respectively), and the median recognition time was twice as long (4 vs. 2 s, respectively). Compared to previous studies, the recognition accuracy of AMD patients in this study was either lower [3,17,21] or similar [17]. Two factors that could result in the observed differences are patient selection and the method of face recognition testing. First, the majority (89%) of patients in this study were in the advanced stage of the disease, which has been shown previously to be associated with the most severely impaired face recognition [3]. Consistent with the advanced disease, most patients also had severely reduced visual acuity, which has also been shown to influence face recognition in previous studies [3,17,21], and it was confirmed in this study. For comparison, the lowest reported VA in the study of Taylor et al. was 0.7 logMAR, while it was 2.0 logMAR in our study. However, the AMD cohort in our study had relatively low recognition accuracy even when comparing AMD cohorts with similar visual acuities [3,21]. At visual acuities around 0.75 logMAR, Barnes et al. reported recognition accuracy between 60 and 80% [21], whereas it was 8–78% in our study (Figure 3A), similar to the findings of Tejeria et al. [17]. These discrepancies are possibly related to the differences in the methodology of face recognition testing. Both studies that reported high recognition accuracy provided a reference image for face-matching [3,21], while the study of Tejeria et al. [17] and the present study performed the test without a reference image, making the test significantly more difficult. The latter approach is probably closer to the real-world situation where a person randomly meets a previously known person and more accurately reveals the true degree of the face recognition difficulty. However, when reference images are not used, caution needs to be taken in the face selection process to exclude any additional factors that could affect recognition. For example, when we excluded two famous people, who became famous relatively recently and might potentially not have been known to a person with a long-standing visual loss, the median recognition accuracy of the AMD group increased by a few percent (from 42% to 45%). In addition to recognition accuracy, our study also measured recognition time, which is another important factor affecting patients’ performance in the real world. The recognition time in the AMD cohort was between 2 and 9 s and 1 and 4 s in control group (median age 68 years). In comparison, Barnes et al. reported a recognition time for their AMD cohort between 3 and 20 s and 3 and 7 s in the control group (median age 73 years) [21]. Both studies demonstrate that the recognition time is significantly reduced in AMD patients; however, the time for all participants was shorter in our study. Since the recorded recognition time contains not only the participant’s recognition time but also the duration from recognition to verbal answer and the reaction time of the researcher, the true recognition time may be even lower and would be most accurately estimated using a device measuring brain responses. Considering the control data, it is possible to conclude that AMD patients, at least in the advanced stage, need approximately twice as long for face recognition than healthy people. Age has been shown to influence face recognition ability. Significant differences in recognition accuracy as well as time were observed between control groups with median ages of 26 and 73 years [21]. In our study, the controls were relatively younger than AMD patients (median 68 vs. 83 years); however, it appears that the age difference was not large enough to importantly affect the results of the study since the statistical analysis on a subset of participants with the same age range was consistent with the original findings. Nevertheless, it is of importance to consider age in the future studies when selecting control subjects.

### 4.2. Insights into Face Recognition Process from the Eye-Tracking Data

In both participant groups, the fixations clustered mainly in the triangle of the eyes, nose, and mouth, which have been established previously as the regions most important for face recognition [8,9,10,11,12]. Furthermore, the nasal area was the most frequent starting point in both groups, which is also consistent with the literature [9]. Although the classical triangle is the cornerstone in face recognition, it has been observed previously that each person has a specific fixation pattern within the triangle, which could be considered a behavioural trait or signature [12]. In this study, we have also observed qualitatively similar fixation patterns in some participants from both groups; however, this is difficult to quantify. Seiple et al. has reported that patients with central visual loss often fixate on the external facial features, such as the chin, outline of the face, and hairline, and this trend has also been observed in the present study. One purpose behind external fixations could also be positioning the face outside of the scotoma (as demonstrated in Figure 4); however, we were not able to confirm a relationship between the use of external fixation and recognition accuracy. The importance of external fixations has been studied by Bernard et al. by selectively deleting either internal or external facial features, which showed that patients with central visual loss were better at recognizing faces using external features [22]. However, the study was performed on a very small cohort (five patients with central visual loss, of those three with AMD) using pre-selected faces that were very recognizable to AMD patients. Further studies may be needed to fully understand the cause and/or benefit of fixations on the external facial features observed in patients with central visual loss. Except for the higher frequency of external fixations, the AMD patients retained the two classical attributes of the face recognition process, i.e., focusing on the nose–eyes–mouth triangle and starting the observation in the nasal area. It is most likely that AMD patients are still able to recognize distinct facial features but have difficulties combining them into a known face. Since it has been observed previously that each person has a specific fixation pattern, which could be considered a behavioural trait or even a signature [12], there is also a possibility that the pattern of fixation in AMD patients is a previously established habit that remained even after visual loss. This could be investigated in the future by asking patients to intentionally look at a specific area of the face.

### 4.3. The Impact of Visual Acuity and Geographic Atrophy Diameter on Face Recognition

There was a significant correlation between VA on the better-seeing eye and face recognition ability in the AMD cohort (Figure 3), consistent with previous observations [3,17,21]. Loss of visual acuity is related to the loss of foveal photoreceptors that provide high-resolution viewing, which is supported by the statistically higher VA in the subgroup of AMD patients with foveal preservation. In accordance, patients with structurally preserved foveal photoreceptors had significantly higher face recognition accuracy in this and previous studies [3]. Taylor et al. has shown that the geographic lesion area also influences recognition accuracy [3]. Although we observed a similar trend in our study, the correlation was not statistically significant. This may be due to a low number of patients but could also suggest that visual acuity and/or a preserved fovea are more important for face recognition than the size of the geographic atrophy. This is also shown in Figure 4, where between two patients with a similar GA diameter, only the one with a preserved fovea, accompanied by better visual acuity, was able to recognize the presented face.

### 4.4. Dementia and AMD

The dementia screening test (MoCA) in many AMD patients showed relatively low scores indicating an increased risk of dementia, which is consistent with observations of higher dementia prevalence among AMD patients [19]; however, unfortunately, the test was not performed in the control group. There was a trend of lower face recognition ability in patients with a lower MoCA score (Figure 8); however, the association was not statistically significant, possibly due to the low number of participants limiting the statistical power. Similarly, Ferris S. et al. could also not conclude that there was a correlation between dementia and face recognition [23]. Other studies on AMD patients and face recognition have unfortunately not studied the effect of dementia but have rather used it as an exclusion factor. For example, Seiple et al. used a low score on a Mini Mental Status Examination, a test comparable to MoCA, as an exclusion criteria [8]. Since previous studies have shown that dementia affects facial memory [20], it is important to include dementia screening in further studies on face recognition and other visual tasks, especially in patients with AMD, to fully determine the potential additional effect of dementia on visual performance.

### 4.5. Study Strengths and Limitations

One of the key strengths of the study is the use of the novel, user-friendly device combining a VR headset with eye-tracking sensors. This method allows recording of real-world recognition patterns, which is something that is completely new as far as AMD is concerned. With this novel technology, we gathered several variables not included in most of the previous studies, such as recognition time and fixation patterns, and gained valuable insight into the process of face recognition in AMD patients. We also included other aspects, such as dementia, that could affect visual perception and not only the aspects that affect vision itself. The main limitation of the study is the small number of participants, which limits the statistical power. There were several observed correlation trends that would possibly be statistically significant in a larger cohort, e.g., the correlation between face recognition accuracy and the GA diameter and the MoCA score. Another limitation of the study is the significant difference in age between AMD and control groups; however, the statistical analysis on age-matched subgroups did not show a significant effect of age in this age category. One limitation of the VR headset is that refractive correction is not typically included. We minimized this disadvantage by excluding subjects with important distance refractive error (see Methods 2.1.), while it was thought that not using correction for near-distance provided a good representation of the real-world situation, where most people do not wear near-vision correction when interacting with others at the distance of 1 m. Custom corrective lenses for the VR headset can be purchased separately and could improve this methodology in the future. A known limitation of this and other face-recognition studies is the difficulty in selection of the famous faces. Care should be taken to select faces that should be recognizable to the tested participants. We have tried our best to select famous people recognizable to participants aged > 50 years and used the control group for confirmation of their recognizability. However, in a patient with a long-lasting visual loss, it is possible that a currently famous person has not been famous yet at the time that visual loss occurred. Although this did not significantly affect the results of the study, it warrants caution in face selection in the future. Since face recognition loss and reading ability loss represent the most important deficits in AMD patients, it would be of interest to explore the relationship between the two disabilities in the future studies. Another parameter that might be considered in the future is the use of 3D or even 4D (i.e., changing with time) faces. A study that compared 2D and 3D recognition showed that 3D faces are recognized more accurately and faster than 2D faces [24]. Furthermore, the fourth dimension, time, has been shown to influence higher-order perceptions such as identity, gender, ethnicity, emotion, and personality [25].

## 5. Conclusions

The study used a novel methodology to study one of the major real-world problems faced by AMD patients. It showed that AMD patients with advanced disease experienced significant difficulties with face recognition, consistent with previous studies exploring face recognition accuracy and providing novel information on recognition time. Furthermore, data gathered from the integrated eye-tracking sensors suggested that AMD patients retained the two classical characteristics of the face recognition process, i.e., fixations mainly in the nose–eyes–mouth triangle and starting observation in the nasal area. Besides that, they displayed a significantly higher frequency of fixations on the external regions of the face; however, without a notable effect on recognition accuracy. The study demonstrates the usability of a VR headset with eye tracking in studying visual perception which could be applicable in the design of clinical studies.

## Figures and Tables

**Table 1:** Demographic characteristics, clinical data, and the results of face recognition testing of AMD patients.

ID	Age (Years)	Sex	Recognition Accuracy (%)	Recognition Time (s)	Freq. of External Fixations (%)	VA (logMAR)		AMD Stage		GA Diameter (Degrees)		Spared Foveal Photoreceptors		MoCA Score
						RE	LE	RE	LE	RE	LE	RE	LE	
1	91	F	0	N/A	61.5	2.0	2.0 *	4a	4a	13.3	10.9	N	N	16
2	89	M	23.1	9.0	N/A	1.3	1.3 *	4a	4b	12.8	N/A	N	N	11
3	79	F	7.7	4.0	100.0	1.0 *	1.0	4a	4a	N/A	N/A	N	N	15
4	87	F	30.8	4.5	100.0	1.0 *	1.7	4b	4b	N/A	N/A	N	N	20
5	68	F	53.9	4.0	N/A	1.0 *	2.0	4b	4b	N/A	N/A	N	N	N/A
6	86	F	46.2	2.5	100.0	0.7 *	2.0	4a	4a	14.2	11.9	N	N	16
7	76	F	46.2	5.5	N/A	1.8	0.5 *	4b	4a	N/A	10.3	N	Y	N/A
8	70	M	46.2	3.5	N/A	0.3 *	0.8	4b	4a	N/A	14.9	N	N	N/A
9	90	F	84.6	4,5	100.0	0.5 *	1.7	4b	4a	N/A	9.0	Y	N	19
10	83	F	30.8	2.5	30.8	1.3	0.4 *	4b	4b	N/A	N/A	N	Y	16
11	82	M	46.2	3.5	84.6	1.7	0.3 *	4b	4b	N/A	N/A	N	Y	14
12	76	M	23.1	4.0	23.1	0.8 *	1.7	4a	4b	7.9	N/A	N	N	N/A
13	75	F	100	2.0	46.2	1.3	0.3 *	3	3	N/A	N/A	Y	Y	18
14	79	M	46.2	5.0	N/A	0.4	0.3 *	4b	2	N/A	N/A	Y	Y	N/A
15	75	F	76.9	3.0	46.2	1.0 *	1.7	4a	4a	7.3	8.6	N	N	19
16	86	F	7.7	7.0	15.4	1.7	0.8 *	4b	4a	N/A	10.7	N	N	18
17	89	M	0.0	N/A	76.9	1.7	1.7 *	4a	4a	17.0	12.7	N	N	N/A
18	85	M	38.5	6.0	92.3	2.3	1.3 *	4b	4b	N/A	N/A	N	N	16

* Better-seeing eye. AMD stages: 2—early; 3—intermediate; 4a—advanced (GA); 4b—advanced (neovascular). Recognition accuracy was calculated as the percentage of recognized faces. Frequency of external fixations was calculated as the percentage of faces on which external fixations were noted. GA—geographic atrophy; MoCA—Montreal Cognitive Assessment; N/A—not applicable/not available; N—no; Y—yes.

**Table 2:** Frequency of different fixation areas during the first five fixations on a representative face (Face 3).

	Proportion of Patients That Fixated First on the Area (%)		Proportion of Patients That Included the Area in Their Initial 5-Fixation Sequence (%)	
Fixation Area	AMD (N = 14)	Controls (N = 16)	AMD (N = 14)	Controls (N = 16)
Face border, forehead, ears	7%	6%	57% *	13% *
Eyes and near the eyes	43%	31%	64%	75%
Nose or near the nose	50%	50%	86%	100%
Mouth or near the mouth	0%	6%	50%	88%
Chin	0%	6%	29%	19%

The regions are listed in an order from top to bottom of the face. * Significant difference.

## Data Availability

Data are available upon request.

## Appendix A

Initials and web sources of famous people’s images used for testing (accessed in July 2023):

1. S.C. https://www.themoviedb.org/person/1281072-sebastian-cavazza/images/profiles

2. L.D. https://www.nba.com/player/1629029/luka-doncic

3. Q.E. https://www.theguardian.com/uk-news/2023/jul/21/naming-of-buildings-after-queen-elizabeth-ii-to-be-closely-protected

4. M.G. https://sl.wikipedia.org/wiki/Mario_Galunič

5. R.P. https://nova24tv.si/slovenija/video-kaj-tako-nizko-mnenje-ima-magnifico-o-slovenski-narodnozabavni-glasbi/attachment/20190821-00954376/

6. P.M. https://sl.wikipedia.org/wiki/Petra_Majdič

7. S.M. https://www.mladinska-knjiga.si/avtorji/svetlana-makarovic

8. Z.M. https://arhiv.onaplus.delo.si/zvezdana-mlakar-o-zlorabah-ko-bi-bilo-treba-kaj-reci-sem-bila-tiho

9. M.M. https://vecer.com/svet-znanih/misa-molk-iskreno-o-zali-in-gasperju-skratka-nekaj-je-tu-sfalilo-nek-komunikacijski-sum-se-je-zgodil-10011165

10. D.M. https://onaplus.delo.si/kolumne/desa-muck-patoloska-vsemogocnost-ali-kdo-bo-odgovarjal-za-posledice-poplav/

11. J.P. https://onthewayinfo.com/?p=270

12. P.P. https://zmagovalci.si/zgodbe/peter-prevc/

13. S.Ž. https://www.dnevnik.si/tag/Slavoj%20Žižek
